# Variants Within Genes *EDIL3* and *ADGRB3* are Associated With Divergent Fecal Egg Counts in Katahdin Sheep at Weaning

**DOI:** 10.3389/fgene.2022.817319

**Published:** 2022-03-10

**Authors:** Gabrielle M. Becker, Joan M. Burke, Ronald M. Lewis, James E. Miller, James L. M. Morgan, Benjamin D. Rosen, Curtis P. Van Tassell, David R. Notter, Brenda M. Murdoch

**Affiliations:** ^1^ Department of Animal, Veterinary and Food Sciences, University of Idaho, Moscow, ID, United States; ^2^ USDA, ARS, Dale Bumpers Small Farms Research Center, Booneville, AR, United States; ^3^ Department of Animal Science, University of Nebraska–Lincoln, Lincoln, NE, United States; ^4^ Department of Pathobiological Sciences, School of Veterinary Medicine, Louisiana State University, Baton Rouge, LA, United States; ^5^ Round Mountain Consulting, Fayetteville, AR, United States; ^6^ USDA, ARS, Animal Genomics and Improvement Laboratory, Beltsville, MD, United States; ^7^ Department of Animal and Poultry Sciences, Virginia Tech, Blacksburg, VA, United States

**Keywords:** gastrointestinal nematodes, helminth, parasites, GWAS, hair sheep

## Abstract

Gastrointestinal nematodes (GIN) pose a severe threat to sheep production worldwide. Anthelmintic drug resistance coupled with growing concern regarding potential environmental effects of drug use have demonstrated the necessity of implementing other methods of GIN control. The aim of this study was to test for genetic variants associated with resistance or susceptibility to GIN in Katahdin sheep to improve the current understanding of the genetic mechanisms responsible for host response to GIN. Linear regression and case-control genome-wide association studies were conducted with high-density genotype data and cube-root transformed weaning fecal egg counts (tFEC) of 583 Katahdin sheep. The case-control GWAS identified two significant SNPs (*P*-values 1.49e-08 to 1.01e-08) within introns of the gene adhesion G protein-coupled receptor B3 (*ADGRB3*) associated with lower fecal egg counts. With linear regression, four significant SNPs (*P*-values 7.82e-08 to 3.34e-08) were identified within the first intron of the gene EGF-like repeats and discoidin domains 3 (*EDIL3*). These identified SNPs were in very high linkage disequilibrium (*r* ^*2*^ of 0.996–1), and animals with alternate homozygous genotypes had significantly higher median weaning tFEC phenotypes compared to all other genotypes. Significant SNPs were queried through public databases to identify putative transcription factor binding site (TFBS) and potential lncRNA differences between reference and alternate alleles. Changes in TFBS were predicted at two SNPs, and one significant SNP was found to be within a predicted lncRNA sequence with greater than 90% similarity to a known lncRNA in the bovine genome. The gene *EDIL3* has been described in other species for its roles in the inhibition and resolution of inflammation. Potential changes of EDIL3 expression mediated through lncRNA expression and/or transcription factor binding may impact the overall immune response and reduce the ability of Katahdin sheep to control GIN infection. This study lays the foundation for further research of *EDIL3* and *ADGRB3* towards understanding genetic mechanisms of susceptibility to GIN, and suggests these SNPs may contribute to genetic strategies for improving parasite resistance traits in sheep.

## Introduction

Gastrointestinal nematodes (GIN) are arguably considered to be one of the greatest health and economic threats to small ruminant production worldwide (Karrow et al., 2014; Escribano et al., 2019; Hassan et al., 2019; Chitneedi et al., 2020). GIN infection contributes to substantial economic losses in both meat and dairy production (Charlier et al., 2020). Symptoms of infection vary depending on the species present, but may include diarrhea, anemia or hypoproteinemia; in extreme cases, high parasite burden can lead to animal death even within the prepatent period of infection (Zajac 2006; Emery et al., 2016). GIN can have substantial repercussions on animal production even when infections are not fatal, as animals experience weight loss and/or poor growth and fail to meet their production potential (Haehling et al., 2020).

The lifecycles of GIN parasites are similar and can be divided between a free-living environmental stage and a parasitic stage within the specific gastrointestinal niche. Sheep that are susceptible to GIN may harbor thousands of worms and contribute to the continued contamination of parasite eggs into the environment (McRae et al., 2015); depending on the helminth species, individual female worms may produce between 100–15,000 eggs per day (Roeber et al., 2013; Emery et al., 2016). Lambs tend to be the most susceptible to severe disease from GIN infection, as their immune systems are not fully mature and have not been previously exposed to parasite antigen (Miller and Horohov 2006; Greer and Hamie 2016). Over time and exposure, sheep tend to become more tolerant of GIN infection (McRae et al., 2015; Benavides et al., 2016). For this reason, young animals are frequently used when examining potential genetic differences in susceptibility and resistance to GIN parasites.

Hair sheep are generally considered to be more tolerant of GIN infection than conventional wool breeds and are capable of maintaining higher packed cell volumes and lower fecal egg counts (FEC) during parasite challenge (Wildeus 1997; Notter et al., 2003; Vanimisetti et al., 2004; González-Garduño et al., 2013). The Katahdin are a composite breed of hair sheep that were founded through crosses of the St. Croix Caribbean hair sheep to temperate wool breeds such as the Suffolk and Wiltshire Horn (Wildeus 1997). Similar to the St. Croix, Katahdin sheep have been described as having increased parasite resistance traits in comparison to wool breeds (Burke and Miller 2004; Vanimisetti et al., 2004; Ngere et al., 2018). Since their development in the 1950s, the Katahdin have become an economically important breed in the United States (Thorne et al., 2021).

Antiparasitic drugs have been the classic approach to help control GIN infection in sheep since their inception. This approach has been impacted in recent years by the growing incidence of anthelmintic drug resistance among GIN parasites (Papadopoulos et al., 2012; Ploeger and Everts 2018; Bosco et al., 2020; Stewart et al., 2020). Selective breeding for parasite resistance is one method that can be utilized to both lessen reliance on pharmaceutical use and improve sheep health and production (McRae et al., 2014; Keane et al., 2018; Aboshady et al., 2020). Parasite resistance can be estimated through several indicator traits, including FEC of parasite eggs per gram of feces, anemia scoring through FAMACHA (FAffa MAlan CHArt) or packed cell volume and measurement of immunoglobulin A (IgA) activity (Davies et al., 2006; Shaw et al., 2012; Ngere et al., 2018; Naeem et al., 2020); of these, FEC is the most common (Ngere et al., 2018). Parasite resistance based on FEC phenotypes has been estimated to be a moderately heritable trait (*h* ^*2*^ of 0.2–0.3), although there are differences in parasite resistance both between and within breeds (Riggio et al., 2013; Brown and Fogarty 2016; Keane et al., 2018).

The current literature suggests that resistance or susceptibility to GIN is likely a polygenic trait controlled by many genes, with each contributing a relatively small overall effect (Kemper et al., 2011; McRae et al., 2014). Genetic markers in genes involved with the innate and adaptive immune responses, including major histocompatibility complex (MHC) and interferon-γ genes, cytokine signaling, mucin secretion and hemostasis pathways have been previously reported and reviewed (Benavides et al., 2016; Al Kalaldeh et al., 2019; Ahbara et al., 2021; González-Garduño et al., 2021). However, it is unlikely that associated markers identified in one breed will be equally applicable to other breeds of sheep, due in part to the differences in linkage disequilibrium (LD) and allele frequencies between breeds (Benavides et al., 2015).

The aim of this study was to further the current understanding of the genetic basis which may influence parasite susceptibility in Katahdin sheep. To accomplish this, a genome-wide association study (GWAS) was performed with high-density (HD) genotype data and weaning FEC of 583 Katahdin sheep. The reference DNA sequences associated with significant SNPs were queried through public functional databases for putative transcription factor binding sites (TFBSs) and sequence identity to known non-coding RNA. These predictive analyses were conducted in order to evaluate possible functional consequences of significant SNPs towards building a better understanding of the potential genetic mechanisms that differentiate susceptible from resistant sheep.

## Materials and Methods

### Fecal Egg Count Data Collection and Preparation

A total of 583 Katahdins (*n* = 275 male, *n* = 308 female) were used in the current study. To ensure representative exposure to GIN, animals were sampled from 20 different Katahdin flocks located in 12 states across the US (AR, GA, ID, IN, MO, NY, OH, OR, TX, VA, WI and WV) (Supplementary Figure S1). All participating flocks were enrolled in the National Sheep Improvement Program (NSIP) and producers consented to perform animal sampling for research purposes. Animals were not treated with anthelmintics within 30 days of FEC sampling. Sampled animals were born in 2019 (*n* = 282), 2018 (*n* = 209) or 2017 or earlier (*n* = 92). Stool samples were collected directly from the rectum of animals at weaning (70.24 ± 11.04 days of age). Weaning FEC were quantified by a certified parasitology laboratory using the modified McMaster technique (Louisiana State University School of Veterinary Medicine and the Virginia-Maryland College of Veterinary Medicine). From previous work, the GIN infections present on most farms used in the current study were expected to be mixed populations of *H. contortus*, *Trichostrongylus* spp., and to a lesser extent, *Teladorsagia* spp., *Cooperia* spp. and *Oesophagostomum* spp (Notter et al., 2017).

As observed in other studies, the FEC data of this study were not normally distributed (Woolaston and Piper 1996; Doyle and Eady 2001; Pollott and Greeff 2004; Kemper et al., 2011; Al Kalaldeh et al., 2019). Normalcy of FEC data was assessed with the Shapiro-Wilks test, kurtosis and skewness analyses conducted in R (data not shown). As a result, weaning FEC were cube-root transformed to normalize the distribution. Average weaning transformed FEC (tFEC) for all samples was 10.18; the minimum and mode weaning tFEC values were 0 (not detected, *n* = 119), the maximum was 38 (*n* = 1) and the median was 8 (*n* = 23) (Supplementary Figure S2).

### Genotyping and Genome-Wide Association Analyses

Blood samples were collected by the participating sheep producers and stored on blood cards until genotyping. Sample DNA were extracted from blood cards and genotyped with the high-density (HD) Illumina 600 K SNP BeadChip (Illumina Inc., San Diego, CA, USA) comprised of 606,006 markers. All DNA extraction and genotyping were conducted at Neogen Corporation—GeneSeek Operations, Lincoln, NE, USA. DNA was extracted by Neogen using the MagMAX™ DNA Multi-Sample Ultra Kit (Thermo Fisher Scientific Cat. A25598).

All samples had a genotypic call rate (CR) > 90%: genotype markers with GenCall score <0.15 were initially excluded, followed by non-autosomal markers, markers with CR < 90%, minor allele frequency <1% or Hardy-Weinberg Equilibrium P-value < 1.0e-06. Redundant markers were filtered such that the marker with the highest CR per genomic location was retained for analyses. Following quality control, 505,914 high-quality autosomal markers and 583 samples were used for analyses.

Association analyses were conducted through SNP and Variation SuiteTM v8.9.0 (Golden Helix, Inc., Bozeman, MT, ww.goldenhelix.com) using the Efficient Mixed-Model eXpedited (EMMAX) (Kang et al., 2010) with additive, dominant and recessive inheritance models as linear regression (LR) or case-control (CC), with cases defined as animals with undetected FEC (*n* = 119). In all tests a genomic relationship matrix was fitted as a random effect and variables with significant relationship to tFEC were fitted as fixed effects. These variables included birth month, birth/rear type (single birth/single reared, twin birth/twin reared, triplet birth/triplet reared, quadruplet birth/quadruplet or triplet reared, quadruplet or triplet birth/twin or single reared) and flock; year was also significant (Supplementary Figure S2) but was not included due to multicollinearity with other variables.

Genome-wide significance was determined by adaptive permutation testing through plink v1.07 with the flags --assoc and --aperm. Each SNP underwent a minimum of 10 permutations and up to 1,000,000 permutations (Purcell et al., 2007, https://zzz.bwh.harvard.edu/plink/perm.shtml#aperm). Other parameters included alpha (0), beta (0.001), intercept (1) and slope (0.001). Permutation testing achieved a minimum empirical *P*-value of 1.00e-06; therefore, genome-wide significance was set to *P*-values < 1.00e-06. Any significant SNPs identified were further analyzed with the Kruskal-Wallis test (weaning tFEC ∼ SNP) and visualized with the package ggpubr in R version 3.6.3 (Hollander and Wolfe 1973; R Core Team 2020; Kassambara 2020).

### Linkage Disequilibrium

The linkage disequilibrium (LD) of significant SNPs identified in the LR recessive model were evaluated. LD was estimated using the composite haplotype method in the SNP and Variation SuiteTM v8.9.0 software (Weir 1996; Golden Helix, Inc., Bozeman, MT, ww.goldenhelix.com). LD was reported using the *r* ^*2*^ statistic as it is thought to be more robust than the *D’* statistic and is generally more favored in the context of association studies (Ardlie et al., 2002; Kijas et al., 2014).

### Non-Coding RNA Prediction Analysis

The significant SNPs positioned within RNA-seq track reads observed in NCBI were investigated against known RNA sequences. The 1,000 bp surrounding each significant SNP in the reference genome (Oar_rambouillet_v1.0) were queried through RNAcentral with the SNP reference and alternative allele (The RNAcentral Consortium, 2019). Due to the position of the SNP within the RNA-seq reads, rs416102123 was at position 501, rs413712238 was at position 729 and rs417983470 was at position 224 of the respective query sequences.

### Transcription Factor Binding Site Prediction

The search tool ConSite was used to analyze input sequences against a TFBS profile collection drawn from the JASPAR database (Sandelin et al., 2004; Fornes et al., 2020). For each significant SNP identified from the LR recessive GWAS, the reference and the alternate alleles were queried along with the 15 nucleotides immediately 3′ and 5′ of the SNP in the reference genome assembly (Oar_rambouillet_v1.0), for a total input of 31 bp. The scores of each putative TFBS were examined to identify potential differences between the reference and alternate allele for each SNP.

## Results

### Genome-Wide Association Analyses

Genome-wide association analyses were performed with HD genotypes and weaning tFEC data of Katahdin sheep. The LR and CC analyses used a mixed-model GWAS to investigate additive, dominant and recessive inheritance models. Quantile-quantile (QQ) plots were constructed for each model to visualize the deviation of observed *P*-values from expected *P*-values. The QQ plots of the recessive models indicated the best control of type I/type II error (Supplementary Figure S3).

**FIGURE 1:**
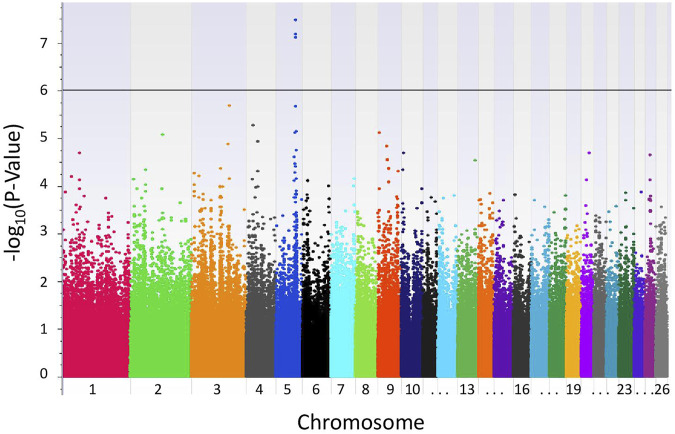
Results of GWAS of Katahdin sheep with weaning tFEC. Manhattan plot from recessive EMMAX model with genome-wide significance threshold defined by P-values <1.0e-06 (black line).

With the LR recessive inheritance model, four significant SNPs were identified within the first intron of the gene epidermal growth factor (EGF)-like repeats and discoidin domains 3 (*EDIL3*), also known as developmental endothelial locus-1 (*DEL-1*) (Figure 1). All four significant SNPs were near to one another, with a minimum distance of 6.3 kbp and maximum distance of 30.8 kbp between SNPs. The SNPs rs405327900 and rs413712238 had the smallest and identical *P*-values (*P*-value = 3.34e-08), followed by rs417983470 (*P*-value = 6.66e-08) and rs416102123 (*P*-value = 7.82e-08). The alternate alleles of significant SNPs identified were observed at a frequency of 35% in this study population. The proportion of weaning tFEC phenotypic variance explained by significant SNPs were 5.4%, 5.4%, 5.2%, and 5.1%, respectively (Table 1).

**TABLE 1:** Significant SNPs from linear regression and case-control GWAS. Table displaying information for significant SNPs identified in linear regression EMMAX GWAS with weaning tFEC phenotypes and recessive, dominant or additive inheritance models and case-control EMMAX GWAS with a recessive inheritance model. A, additive; CC, case-control; Chr, chromosome; D, dominant; FDR, false discovery rate; LR, linear regression; MAF, minor allele frequency; PVE, proportion of variance explained; R, recessive.

Model	Chr	rs Number	Position bp	*P*-Value		MAF	PVE	Gene
				Unadjusted	FDR			
LR-R	5	rs405327900	88,129,857	3.34e-08	0.0169	0.3532	0.0539	*EDIL3*
	5	rs413712238	88,154,411	3.34e-08	0.0084	0.3511	0.0539	
	5	rs417983470	88,160,689	6.66e-08	0.0112	0.3511	0.0516	
	5	rs416102123	88,144,942	7.82e-08	0.0099	0.3500	0.0511	
LR-A	2	rs428768700	14,076,309	1.49e-08	0.0075	0.0214	0.0567	*PALM2AKAP2*
LR-D	2	rs428768700	14,076,309	1.49e-08	0.0075	0.0214	0.0567	*PALM2AKAP2*
	10	rs417380632	6,660,635	2.32e-07	0.0588	0.3671	0.0475	24 kpb 5′ of *PCDH17*
CC-R	9	rs416881989	5,046,485	1.01e-08	0.0051	0.4218	0.0580	*ADGRB3*
	9	rs421657777	5,325,635	5.81e-08	0.0147	0.4786	0.0521	

The results of the LR additive and dominant GWAS models were similar. Both of these models identified the significant SNP rs428768700 with *P*-value = 1.49e-08 and 5.7% proportion of variance explained. This SNP is positioned within the first intron of the PALM2 and AKAP2 fusion gene (*PALM2AKAP2*). The annotation of this gene is unclear, as PALM2 and AKAP2 have been previously annotated as separate genes (NCBI Resource Coordinators 2016). No animals were homozygous for the alternate allele at this marker: 25 animals were heterozygous and the remaining 558 animals were homozygous for the reference allele. The dominant model identified an additional significant SNP, rs417380632, with *P*-value = 2.32e-07 and 4.8% proportion of variance explained. This SNP is located approximately 24 kbp before the start of the gene protocadherin 17 (*PCDH17*) (Table 1).

**FIGURE 2:**
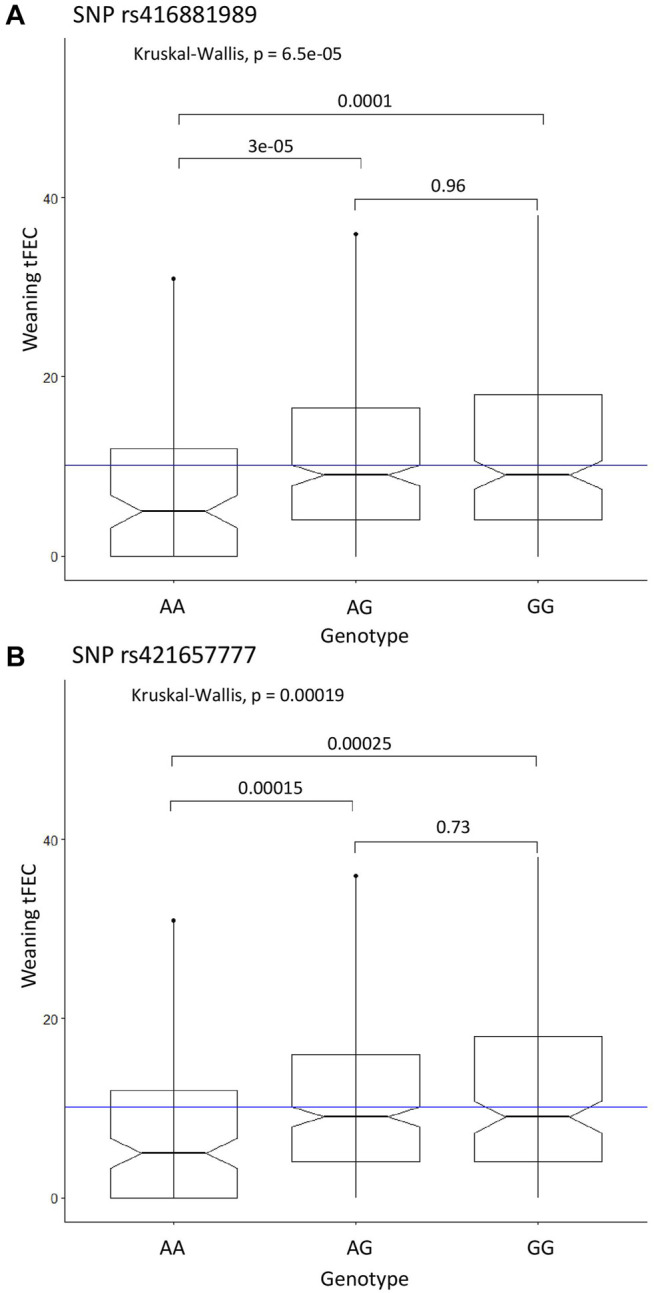
Significant SNP genotypes and weaning tFEC phenotypes from the case-control GWAS. **(A)** Kruskal-Wallis results for SNP rs416881989, **(B)** Kruskal-Wallis results for SNP rs421657777. The mean tFEC value is represented by the blue line.

The recessive CC model identified two significant SNPs on chromosome 9: rs416881989 with *P*-value = 1.01E-08 and 5.8% proportion of variance explained and rs421657777 with *P*-value = 5.81E-08 and 5.2% proportion of variance explained (Table 1). These markers are located 279 kbp apart and are within the gene adhesion G protein-coupled receptor B3 (*ADGRB3*). Marker rs421657777 is positioned within intron 17 and marker rs416881989 is positioned within intron 26 of *ADGRB3*. Although G protein-coupled receptors are involved in many physiological processes (Suchý et al., 2020), little is known about the specific role of adhesion G protein-coupled receptors in the sheep immune response. Sheep with homozygous alternate genotypes (AA) at these markers had significantly lower tFEC compared to sheep with heterozygous and homozygous references genotypes (Figure 2).

### Linkage Disequilibrium

Analysis of LD between the four significant SNPs identified in the LR recessive GWAS revealed that SNPs were in near to perfect LD (*r* ^*2*^ = 0.996–1). The LD between SNPs rs405327900, rs413712238 and rs416102123 was *r* ^*2*^ = 1, indicating perfect LD, while the LD of rs417983470 to all others was *r* ^*2*^ = 0.996, indicating high LD. Given the strength of correlations between these SNPs, the frequencies at which these alleles occurred were determined within the sample population. The majority of samples were either homozygous for the alternate alleles (11.8% of samples), homozygous for the reference alleles (41.9% of samples) or were entirely heterozygous (46.1% of samples) at all four SNPs (Table 2). One sample was excluded from frequency calculations as it possessed alternate alleles at three SNPs and a heterozygous genotype at SNP rs417983470. Additionally, eight samples had missing genotypes inferred to be placed into an allele distribution.

**TABLE 2:** Summary of significant SNP genotype frequencies (linear regression GWAS, recessive) in Katahdin study population. The majority of animals examined (*n* = 582) had either entirely homozygous alternate, entirely homozygous reference or entirely heterozygous genotypes at the four significant SNPs. One animal is not represented in the genotype frequency table as it possessed homozygous alternate genotypes with the exception of a single heterozygous genotype at SNP rs417983470. A further eight animals had missing genotypes inferred in order to be placed into allele distributions.

SNP Genotype	rs405327900 (A/C)	rs416102123 (G/A)	rs413712238 (A/G)	rs417983470 (A/G)	Frequency
Homozygous Alternate	CC	AA	GG	GG	0.118
Homozygous Reference	AA	GG	AA	AA	0.419
Heterozygous	AC	GA	AG	AG	0.461

### Kruskal-Wallis Test

**FIGURE 3:**
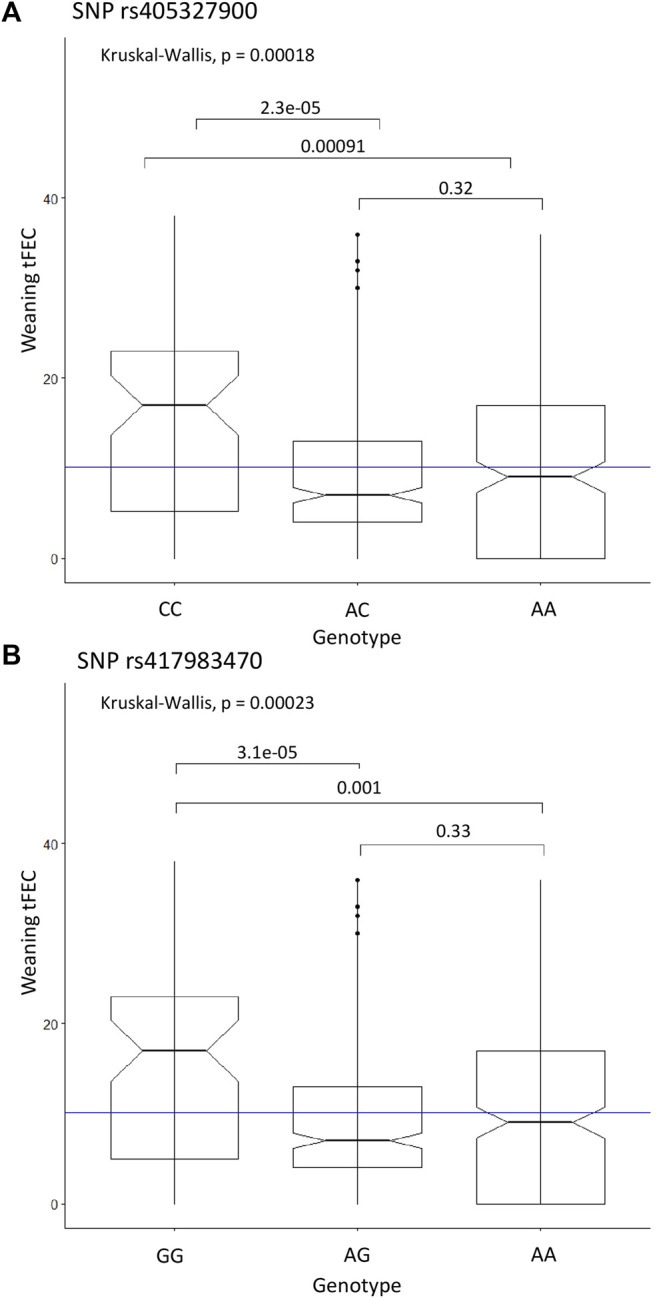
Significant SNP genotypes and weaning tFEC phenotypes from the linear regression recessive GWAS. **(A)** Kruskal-Wallis results for SNP rs405327900 represents rs416102123 and rs413712238 as well due to perfect LD, **(B)** Kruskal-Wallis results for SNP rs417983470. The mean tFEC value is represented by the blue line.

The Kruskal-Wallis test was used as a non-parametric analysis of variance to examine weaning tFEC phenotypes between genotypes of significant SNPs. Due to LD (*r* ^*2*^ = 1), SNPs rs405327900, rs416102123 and rs413712238 all exhibited identical Kruskal-Wallis test results, with overall *p* = 0.00018. The homozygous alternate genotypes (CC, AA, GG) were significantly different when compared to heterozygous (AC, GA, AG) (*p* = 2.3e-05) and homozygous reference genotypes (AA, GG, AA) (*p* = 0.00091), respectively. Again based on the Kruskal-Wallis test, with an overall *p* = 0.00023, the homozygous alternate genotype (GG) for SNP rs417983470 significantly differed from the heterozygous (AG) (*p* = 3.1e-05) and homozygous reference (AA) (*p* = 0.001) genotypes. For all four SNPs, the homozygous alternate genotype was found to have a significantly higher median weaning tFEC than all other genotypes (Figure 3).

### Non-Coding RNA Prediction Analysis

**FIGURE 4:**
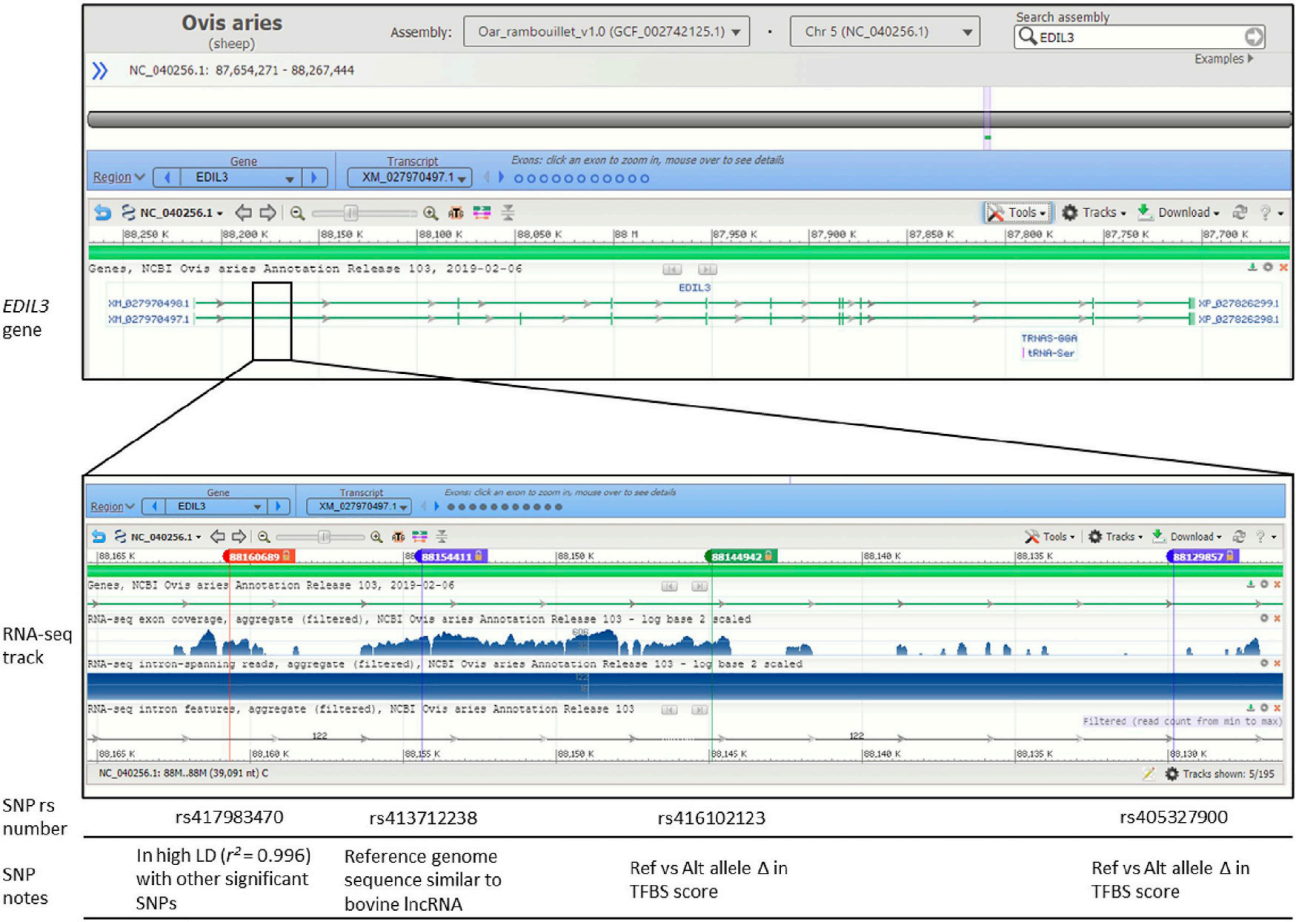
Genomic context of significant SNPs within gene *EDIL3*. Figure displays image from the National Center for Biotechnology Information (NCBI) Genome Data Viewer tool. Genomic context is displayed for the Oar_rambouillet_v1.0 genome assembly. The SNPs rs417983470, rs413712238 and rs416102123 are within NCBI *Ovis aries* Annotation Release 103 RNA-seq reads. Relevant linkage disequilibrium (LD), long non-coding RNA (lncRNA) query and transcription factor binding site (TFBS) prediction results are noted.

**FIGURE 5:**
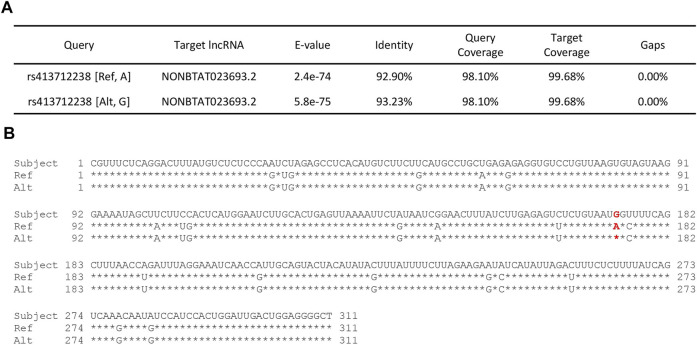
RNAcentral results for SNP rs413712238. **(A)** Table contains a summary of RNAcentral query results in which query SNP sequence matches with ≥90% identity score to known RNA sequence. SNP rs413712238 matched with bovine lncRNA (UMD_3.1 reference genome assembly). **(B)** Full sequence of lncRNA NONBTAT023693.2 (*Bos taurus*) vs. the query reference and alternate allele sequences for rs413712238. The location of SNP rs413712238 is given in red.

Three of the significant SNPs (rs413712238, rs417983470, rs416102123) were located within a region with previously reported RNA-seq reads (NCBI Genome Data Viewer: RNA-seq intron-spanning reads, aggregate (filtered), NCBI *Ovis aries* Anotation Release 103—log base 2 scaled) (Figure 4). These SNPs were investigated for similarity to known non-coding RNA sequences curated by the RNAcentral database. This analysis identified that SNP rs413712238 was located within a target RNA sequence which achieved an identity score >90% (Figure 5A). The sequence identified is that of NONBTAT023693.2, a lncRNA 311 nucleotides in length in the *Bos taurus* genome (*Bos*_taurus_UMD_3.1/bosTau6 assembly). The reference allele query matched at 289 positions and the alternate allele query matched at 290 positions of the target RNA sequence (Figure 5B).

### Transcription Factor Binding Site Prediction

To investigate the potential consequences of *EDIL3* intronic variants, significant SNPs were examined for putative TFBSs. The SNPs rs405327900, rs413712238 and rs416102123 were located within predicted TFBS sequences. TFBS matrix score differences between alternate and reference alleles were observed for two of the three SNPs. The SNP rs405327900 was predicted within the transcription factors T-box transcription factor T (TBXT, also known as brachyury) and proto-oncogene c-Fos (c-Fos). Whereas the alternate allele introduced a TFBS for c-Fos which was not present at the reference allele, the binding score prediction for TBXT was greater with the reference allele compared to the alternate allele. Regarding SNP rs416102123, a sequence of 10 nucleotides matched with transcription factors belonging to the Rel/NF-κB family: Rel class, proto-oncogene c-Rel (c-Rel) and transcription factor p65 (also known as RelA). These TFBS were predicted to have greater scores with the alternate allele vs. reference allele. A TFBS for interferon regulatory factor 1 (IRF1) was identified with rs416102123 reference and lost with alternate allele sequence (Table 3, Supplementary Figure S4). Matrix scores of all predicted TFBS results are reported in Supplementary Table S1.

**TABLE 3:** Results from ConSite transcription factor binding site (TFBS) analysis showing differences between reference and alternate allele sequences. SNPs rs405327900 and rs416102123 had predicted TFBS score differences between their reference and alternate alleles. (*) denotes transcription factor is only present in alternate allele sequence, (＾) denotes transcription factor is only present in reference allele sequence.

**Transcription Factor**	**Score difference Alt vs. Ref**
rs405327900	
TBXT/Brachyury	−2.308
c-Fos*	+8.141
rs416102123	
REL class	+0.148
c-Rel	+0.844
p65/RelA	+1.310
IRF1^	−10.652

## Discussion

This GWAS is the first to identify significant associations with the gene *EDIL3* and sheep response to GIN infection. This study capitalized on the increased resolution of the HD genotype array to detect associations within a relatively large sample size. Several prior studies have reported quantitative trait loci (QTL) within sequence near *EDIL3*, including Sallé et al. (2012), Kemper et al. (2011), Becker et al. (2020). These studies reported SNPs of significance or suggestive significance positioned 3.7 Mb 5′ (rs401461177), 7.1 Mb 5′ (rs415558729) and 8.7 Mb 3′ (rs55632043) of *EDIL3*, respectively. None of the aforementioned studies tested the four SNPs significant in the present study, as these SNPs are unique to the HD array and could not be investigated in studies that utilize 50K array data.

The gene *EDIL3* encodes the protein epidermal growth factor (EGF)-like repeat and discoidin I-like domain-containing protein 3, a secreted glycoprotein which acts as an integrin ligand and has non-redundant roles in multiple stages of the immune response, including myelopoiesis, anti-inflammatory regulation of neutrophil infiltration and resolution of inflammation (Mitroulis et al., 2017; Chen et al., 2018; Hajishengallis and Chavakis 2019; Li et al., 2021). Endothelial cells secrete EDIL3 to limit neutrophil recruitment to sites of infection and restrain the initiation of inflammation. This is accomplished by interfering with the interaction of lymphocyte function-associated antigen-1 (LFA-1) integrin on leukocytes with intercellular adhesin molecule-1 (ICAM-1) on the surface of vascular endothelial cells (Shin et al., 2013; Li et al., 2021). It is also thought to inhibit ICAM-1-dependent chemokine release (CXCL2 and CCL3) by neutrophils, and EDIL3 is thought to be involved in downstream processes of inflammation through binding to the αvβ3 integrin on the macrophage and phosphatidylserine on the apoptotic neutrophil cell to mediate efferocytosis and inflammation resolution (Hanayama et al., 2004; Kourtzelis et al., 2019; Li et al., 2021). EDIL3 is reciprocally regulated with the proinflammatory cytokine IL-17 (Shin et al., 2013; Choi et al., 2015; Yan et al., 2018; Li et al., 2021). IL-17 is produced by Th17 cells to promote recruitment of macrophages and neutrophils to aggravate chronic inflammation (McRae et al., 2015; Sehrawat and Rouse, 2017). IL-17 functions with TNF-α to enhance expression of neutrophil-attracting chemokines (CXCL1, CXCL2, CXCL5), which leads to an increase in leukocyte transmigration as well as CXCR2-dependent neutrophil transmigration *in vivo* (Griffin et al., 2012). Considering these functions, EDIL3 protein could have potential consequences on the initiation, sustainment and/or resolution of inflammation during GIN parasite infection in sheep.

To investigate the potential role of *EDIL3* markers associated with tFEC phenotypes, the reference genome regions encompassing significant SNPs were investigated for differences between predicted TFBS or ncRNA sequences of reference vs. alternate SNP alleles. Differences in predicted putative TFBS were identified at two SNPs within the first intron of *EDIL3*. These findings are of interest, as transcription mediated by RNA-polymerase II may be activated or repressed by the presence of transcription factors binding in specific regions of DNA (Sandelin et al., 2004). The SNP rs405327900 was predicted within a c-Fos TFBS and SNP rs416102123 was predicted within REL class/c-Rel/p65 TFBS and IRF1 TFBS. The current literature contains well-documented roles for these transcription factors within the immune system (Taki et al., 1997; Foletta et al., 1998; Pahl 1999; Taniguchi et al., 2001; Vallabhapurapu and Karin, 2009; Forero et al., 2019).

Fos proteins enhance DNA-binding activity through the formation of stable heterodimers with Jun proteins (Hess et al., 2004) and members of the Fos and Jun protein families are components of the activator protein-1 (AP-1) transcription factor complex (Hess et al., 2004; Ray et al., 2006; Szalóki et al., 2015). The transcription factors p65 and c-Rel are members of the Rel/NF-κB family (Pahl 1999). These transcription factors are thought to be important in many processes, including: inducing expression of cytokines and chemokines, proteins involved in antigen presentation, cell adhesion molecules, genes involved in stress response, apoptosis and angiogenesis (Ballard et al., 1988; Roebuck 1999; Shaulian and Karin 2002; Eferl and Wagner, 2003; Herrmann et al., 2003; Liang et al., 2004; Gao et al., 2006; Bunting et al., 2007; Son et al., 2008; Reuter et al., 2010). Although these transcription factors do not have confirmed roles within *EDIL3* gene regulation, transcription factor AP-1 has been predicted to target the human *EDIL3* gene according to the MotifMap Predicted Transcription Factor Targets database (Xie et al., 2009; Rouillard et al., 2016).

Importantly, NF-κB and AP-1 are involved in pro-inflammatory pathways. Neutrophil recruitment by bronchial epithelial cells was found to be regulated by NF-κB and AP-1 transcription factors through expression of proinflammatory cytokines (Desaki et al., 2000). Differential activation and promoter binding of AP-1 and NF-κB have been associated with both IL-8 and ICAM-1 gene expression, which regulate transendothelial migration of neutrophils (Roebuck 1999; Desaki et al., 2000; Reuter et al., 2010). The transcription factor interferon regulatory factor 1 (IRF1) is involved in both innate and adaptive immunity (Taniguchi et al., 2001; Honda and Taniguchi 2006; Kano et al., 2008). IRF1 is involved in induction of type 1 interferon genes as well as the activation of interferon-stimulated genes (Henderson et al., 1997). It has been found that T cells from mice lacking IRF1 fail to mount a Th1 response, and instead undergo exclusive Th2 differentiation *in vitro* (Taki et al., 1997).

Long non-coding RNA (lncRNA) expression has been found to correlate with the expression of nearby genes (Ebisuya et al., 2008; Cabili et al., 2011; Engreitz et al., 2016) and lncRNA may regulate miRNA function, thereby influencing gene expression (Ballantyne et al., 2016). lncRNAs play major roles in gene regulation as well as many other biological processes, and the deregulation of lncRNA have been indicated in disease processes (Wu et al., 2014; Dhanoa et al., 2018; Jiang et al., 2019). In this study, the reference sequence surrounding SNP rs413712238 matched with a high identity to a lncRNA described within the bovine genome. Further work is necessary to confirm the presence of lncRNA transcript within ovine *EDIL3* and to explore the potential functional implications.

The significant SNPs identified in this study were in high LD (*r* ^*2*^ = 0.996–1) and genotypes were observed at high frequencies in the study population. Animals that possessed the homozygous alternate genotype at each significant SNP were likely more susceptible to GIN, as indicated by significantly higher median weaning tFEC phenotypes. These animals were predicted to match the lncRNA NONBTAT023693.2 with a greater identity score, possess a TFBS for c-Fos and lack a TFBS for IRF1. Animals with either homozygous reference or heterozygous genotypes were likely less susceptible to GIN infection when compared to animals with homozygous alternate genotypes. Animals that possessed homozygous reference genotypes at each significant SNP had lower predicted identity to NONBTAT023693.2 lncRNA, lacked c-Fos TFBS and possessed IRF1 TFBS. Animals that were heterozygous at significant SNPs possessed a combination of reference and alternate allele genetic attributes, but were phenotypically similar to animals with only reference alleles. These results suggest that the presence of c-Fos TFBS on both strands within *EDIL3*, either in conjunction with or independent of the presence of intronic lncRNA, might influence gene expression towards a more susceptible immune response to GIN. Conversely, the presence of IRF1 TFBS on one or both strands may influence a less susceptible response to GIN.

Based on these results, the expression of EDIL3 appears to contribute to GIN susceptibility in Katahdin sheep, and this expression may be mediated by allelic variants within TFBS and/or lncRNA sequence within the first intron (Figure 4). The evidence presented here supports the hypothesis that *EDIL3*-mediated susceptibility to GIN is recessively inherited. The prediction of c-Fos TFBS within rs405327900 sequence of more susceptible animals and IRF1 TFBS exclusively within rs416102123 sequence of less susceptible animals suggests opposing roles for these transcription factors within the context of *EDIL3* gene regulation. Both c-Fos and IRF1 have been known to promote or suppress expression of target genes, and it is unclear how changes in EDIL3 expression may function in the context of GIN infection in sheep.

Upregulation of Th17-associated genes has been associated with both resistance and susceptibility to GIN in sheep (McRae et al., 2015). The Th17 response has been linked with the inability to control L3 colonization, adult worm infection and egg production during infection by *T. circumcincta* in Blackface lambs (Gossner et al., 2012). Lambs of St. Croix and Barbados Blackbelly descent mounted a stronger Th17 response during *H. contortus* challenge compared with more susceptible composite wool lambs (MacKinnon et al., 2009), and genetic loci related to Th17 genes were recently associated with Florida Native sheep resistance to *H. contortus* (Estrada-Reyes et al., 2019).

It may be hypothesized that suppression of *EDIL3* gene transcription by either c-Fos or IRF1 may have respectively harmful or beneficial effects on GIN infection through reciprocal upregulation of IL-17 and the Th17 response. The precise effects of a Th17 response likely depends upon the breed of sheep and predominant parasite species involved in the infection. Additionally, deficiency of EDIL3 in mice has been shown to activate transforming growth factor *β* (TGF-β); upregulation of TGF-β may be associated with an effective immune response to GIN in mammalian hosts (Ahbara et al., 2021), and earlier expression of TGF-β and IL10 have been associated with resistant compared to susceptible Morada Nova sheep (Toscano et al., 2019). Further research is necessary to understand what involvement *EDIL3* may have on the immune response to GIN in Katahdin sheep.

In this study, four SNPs were identified within the first intron of *EDIL3* that were significantly associated with weaning tFEC in Katahdin sheep. Furthermore, animals with alternate homozygous genotypes at significant SNPs were found to have significantly greater median weaning tFEC phenotypes, indicating that these genotypes incur greater risk for susceptibility to GIN. Significant SNPs may have functional consequences through altered TFBS and/or lncRNA sequence, thereby affecting *EDIL3* gene expression. Further work is needed to elucidate causative variants and precise functional mechanisms as well as to confirm the presence of predicted TFBS and lncRNA. To the best of our knowledge, this is the first study to associate the immune gene *EDIL3* with response to parasites in sheep.

## Data Availability

The datasets presented in this study can be found in the website of The Animal-Germplasm Resources Information Network (Animal-GRIN): https://agrin.ars.usda.gov/main_webpage_dev/ars.

## Abbreviations

AR, Arkansas; GA, Georgia; ID, Idaho; IN, Indiana; MO, Missouri; NY, New York; OH, Ohio; OR, Oregon; TX, Texas; VA, Virginia; WI, Wisconsin; WV, West Virginia.

## References

[B1] Aboshady H. M. Stear M. J. Johansson A. Jonas E. Bambou J. C. (2020). Immunoglobulins as Biomarkers for Gastrointestinal Nematodes Resistance in Small Ruminants: A Systematic Review. Sci. Rep. 10 (1), 7765. 10.1038/s41598-020-64775-x 32385321 PMC7210940

[B2] Ahbara A. M. Rouatbi M. Gharbi M. Rekik M. Haile A. Rischkowsky B. (2021). Genome-wide Insights on Gastrointestinal Nematode Resistance in Autochthonous Tunisian Sheep. Sci. Rep. 11 (1), 9250. 10.1038/s41598-021-88501-3 33927253 PMC8085236

[B3] Al Kalaldeh M. Gibson J. Lee S. H. Gondro C. van der Werf J. H. J. (2019). Detection of Genomic Regions Underlying Resistance to Gastrointestinal Parasites in Australian Sheep. Genet. Sel Evol. 51 (1), 37. 10.1186/s12711-019-0479-1 31269896 PMC6609385

[B4] Ardlie K. G. Kruglyak L. Seielstad M. (2002). Patterns of Linkage Disequilibrium in the Human Genome. Nat. Rev. Genet. 3, 299–309. 10.1038/nrg777 11967554

[B5] Ballantyne M. McDonald R. Baker A. (2016). lncRNA/MicroRNA Interactions in the Vasculature. Clin. Pharmacol. Ther. 99 (5), 494–501. 10.1002/cpt.355 26910520 PMC4881297

[B6] Ballard D. Böhnlein E. Lowenthal J. Wano Y. Franza B. Greene W. (1988). HTLV-I Tax Induces Cellular Proteins that Activate the Kappa B Element in the IL-2 Receptor Alpha Gene. Science 241 (4873), 1652–1655. 10.1126/science.241.4873.1652 2843985

[B7] Becker G. M. Davenport K. M. Burke J. M. Lewis R. M. Miller J. E. Morgan J. L. M. (2020). Genome‐wide Association Study to Identify Genetic Loci Associated with Gastrointestinal Nematode Resistance in Katahdin Sheep. Anim. Genet. 51 (2), 330–335. 10.1111/age.12895 31900974 PMC7064973

[B8] Benavides M. V. Sonstegard T. S. Kemp S. Mugambi J. M. Gibson J. P. Baker R. L. (2015). Identification of Novel Loci Associated with Gastrointestinal Parasite Resistance in a Red Maasai X Dorper Backcross Population. PloS one 10 (4), e0122797. 10.1371/journal.pone.0122797 25867089 PMC4395112

[B9] Benavides M. V. Sonstegard T. S. Van Tassell C. (2016). Genomic Regions Associated with Sheep Resistance to Gastrointestinal Nematodes. Trends Parasitology 32 (6), 470–480. 10.1016/j.pt.2016.03.007 27183838

[B10] Bosco A. Kießler J. Amadesi A. Varady M. Hinney B. Ianniello D. (2020). The Threat of Reduced Efficacy of Anthelmintics against Gastrointestinal Nematodes in Sheep from an Area Considered Anthelmintic Resistance-free. Parasites Vectors 13 (1), 457. 10.1186/s13071-020-04329-2 32907633 PMC7487796

[B11] Brown D. J. Fogarty N. M. (2017). Genetic Relationships between Internal Parasite Resistance and Production Traits in Merino Sheep. Anim. Prod. Sci. 57, 209–215. 10.1071/AN15469

[B12] Bunting K. Rao S. Hardy K. Woltring D. Denyer G. S. Wang J. (2007). Genome-Wide Analysis of Gene Expression in T Cells to Identify Targets of the NF-Κb Transcription Factor C-Rel. J. Immunol. 178 (11), 7097–7109. Baltimore, Md. 1950. 10.4049/jimmunol.178.11.7097 17513759

[B13] Burke J. M. Miller J. E. (2004). Relative Resistance to Gastrointestinal Nematode Parasites in Dorper, Katahdin, and St. Croix Lambs under Conditions Encountered in the southeastern Region of the United States. Small Ruminant Res. 54 (1-2), 43–51. 10.1016/j.smallrumres.2003.10.009

[B14] Cabili M. N. Trapnell C. Goff L. Koziol M. Tazon-Vega B. Regev A. (2011). Integrative Annotation of Human Large Intergenic Noncoding RNAs Reveals Global Properties and Specific Subclasses. Genes Dev. 25 (18), 1915–1927. 10.1101/gad.17446611 21890647 PMC3185964

[B15] Charlier J. Rinaldi L. Musella V. Ploeger H. W. Chartier C. Vineer H. R. (2020). Initial Assessment of the Economic burden of Major Parasitic Helminth Infections to the Ruminant Livestock Industry in Europe. Prev. Vet. Med. 182, 105103. 10.1016/j.prevetmed.2020.105103 32750638

[B16] Chen L.-S. Kourtzelis I. Singh R. Grossklaus S. Wielockx B. Hajishengallis G. (2018). Endothelial Cell-specific Overexpression of Del-1 Drives Expansion of Haematopoietic Progenitor Cells in the Bone Marrow. Thromb. Haemost. 118, 613–616. Advance online publication. 10.1055/s-0038-1624582 PMC6081267 29415284

[B17] Chitneedi P. K. Arranz J. J. Suárez‐Vega A. Martínez‐Valladares M. Gutiérrez‐Gil B. (2020). Identification of Potential Functional Variants Underlying Ovine Resistance to Gastrointestinal Nematode Infection by Using RNA‐Seq. Anim. Genet. 51 (2), 266–277. 10.1111/age.12894 31900978

[B18] Choi E. Y. Lim J.-H. Neuwirth A. Economopoulou M. Chatzigeorgiou A. Chung K.-J. (2015). Developmental Endothelial Locus-1 Is a Homeostatic Factor in the central Nervous System Limiting Neuroinflammation and Demyelination. Mol. Psychiatry 20 (7), 880–888. 10.1038/mp.2014.146 25385367 PMC4351922

[B19] Davies G. Stear M. J. Benothman M. Abuagob O. Kerr A. Mitchell S. (2006). Quantitative Trait Loci Associated with Parasitic Infection in Scottish Blackface Sheep. Heredity 96 (3), 252–258. 10.1038/sj.hdy.6800788 16391549

[B20] Desaki M. Takizawa H. Ohtoshi T. Kasama T. Kobayashi K. Sunazuka T. (2000). Erythromycin Suppresses Nuclear Factor-Κb and Activator Protein-1 Activation in Human Bronchial Epithelial Cells. Biochem. biophysical Res. Commun. 267 (1), 124–128. 10.1006/bbrc.1999.1917 10623585

[B21] Dhanoa J. K. Sethi R. S. Verma R. Arora J. S. Mukhopadhyay C. S. (2018). Long Non-coding RNA: its Evolutionary Relics and Biological Implications in Mammals: a Review. J. Anim. Sci. Technol. 60, 25. 10.1186/s40781-018-0183-7 30386629 PMC6201556

[B22] Doyle E. K. Eady S. J. (2001). Alternate Approaches to Presentation of Worm Resistance Breeding Values for Australian Sheep. Proc. Assoc. Advmt. Anim. Breed. Genet. 14, 195–198.

[B23] Ebisuya M. Yamamoto T. Nakajima M. Nishida E. (2008). Ripples from Neighbouring Transcription. Nat. Cel Biol 10 (9), 1106–1113. 10.1038/ncb1771 19160492

[B24] Eferl R. Wagner E. F. (2003). AP-1: a Double-Edged Sword in Tumorigenesis. Nat. Rev. Cancercancer 3 (11), 859–868. 10.1038/nrc1209 14668816

[B25] Emery D. L. Hunt P. W. Le Jambre L. F. (2016). *Haemonchus contortus*: the Then and Now, and where to from Here. Int. J. Parasitol. 46 (12), 755–769. 10.1016/j.ijpara.2016.07.001 27620133

[B26] Engreitz J. M. Haines J. E. Perez E. M. Munson G. Chen J. Kane M. (2016). Local Regulation of Gene Expression by lncRNA Promoters, Transcription and Splicing. Nature 539 (7629), 452–455. 10.1038/nature20149 27783602 PMC6853796

[B27] Escribano C. Saravia A. Costa M. Castells D. Ciappesoni G. Riet-Correa F. (2019). Resistance to Haemonchus contortus in Corriedale Sheep Is Associated to High Parasite-specific IgA Titer and a Systemic Th2 Immune Response. Sci. Rep. 9 (1), 19579. 10.1038/s41598-019-55447-6 31862904 PMC6925110

[B28] Estrada-Reyes Z. M. Rae O. Postley C. Jiménez Medrano M. B. Leal Gutiérrez J. D. Mateescu R. G. (2019). Association Study Reveals Th17, Treg, and Th2 Loci Related to Resistance to *Haemonchus contortus* in Florida Native Sheep1. J. Anim. Sci. 97 (11), 4428–4444. 10.1093/jas/skz299 31541548 PMC6827414

[B29] Foletta V. C. Segal D. H. Cohen D. R. (1998). Transcriptional Regulation in the Immune System: All Roads lead to AP-1. J. Leukoc. Biol. 63 (2), 139–152. 10.1002/jlb.63.2.139 9468273

[B30] Forero A. Ozarkar S. Li H. Lee C. H. Hemann E. A. Nadjsombati M. S. (2019). Differential Activation of the Transcription Factor IRF1 Underlies the Distinct Immune Responses Elicited by Type I and Type III Interferons. Immunity 51 (3), 451–464. e6. 10.1016/j.immuni.2019.07.007 31471108 PMC7447158

[B31] Fornes O. Castro-Mondragon J. A. Khan A. van der Lee R. Zhang X. Richmond P. A. (2020). JASPAR 2020: Update of the Open-Access Database of Transcription Factor Binding Profiles. Nucleic Acids Res. 48 (D1), D87–D92. 10.1093/nar/gkz1001 31701148 PMC7145627

[B32] Gao Y. Hannan N. R. F. Wanyonyi S. Konstantopolous N. Pagnon J. Feng H. C. (2006). Activation of the Selenoprotein SEPS1 Gene Expression by Pro-inflammatory Cytokines in HepG2 Cells. Cytokine 33 (5), 246–251. 10.1016/j.cyto.2006.02.005 16574427

[B33] Garza J. J. Greiner S. P. Bowdridge S. A. (2017). Serum‐mediated *Haemonchus contortus* Larval Aggregation Differs by Larval Stage and Is Enhanced by Complement. Parasite Immunol. 39 (3). 10.1111/pim.1240910.1111/pim.12409 28063162

[B34] González-Garduño R. Arece-García J. Torres-Hernández G. (2021). Physiological, Immunological and Genetic Factors in the Resistance and Susceptibility to Gastrointestinal Nematodes of Sheep in the Peripartum Period: A Review. Helminthologia 58 (2), 134–151. 10.2478/helm-2021-0020 34248374 PMC8256458

[B35] González-Garduño R. Mendoza-de Gives P. Torres-Hernández G. (2013). Variability in the Fecal Egg Count and the Parasitic burden of Hair Sheep after Grazing in Nematode Infected Paddocks. Pesq. Vet. Bras. 33 (4), 469–475. 10.1590/s0100-736x2013000400010

[B36] Gossner A. G. Venturina V. M. Shaw D. J. Pemberton J. M. Hopkins J. (2012). Relationship between Susceptibility of Blackface Sheep to Teladorsagia Circumcincta Infection and an Inflammatory Mucosal T Cell Response. Vet. Res. 43 (1), 26. 10.1186/1297-9716-43-26 22455366 PMC3422184

[B37] Greer A. W. Hamie J. C. (2016). Relative Maturity and the Development of Immunity to Gastrointestinal Nematodes in Sheep: an Overlooked Paradigm. Parasite Immunol. 38 (5), 263–272. 10.1111/pim.12313 26989873

[B38] Griffin G. K. Newton G. Tarrio M. L. Bu D.-x. Maganto-Garcia E. Azcutia V. (2012). IL-17 and TNF-α Sustain Neutrophil Recruitment during Inflammation through Synergistic Effects on Endothelial Activation, J.I., 188(12). Baltimore, Md, 6287–6299. 10.4049/jimmunol.1200385 PMC3370121 22566565

[B39] Haehling M. B. Cruvinel G. G. Toscano J. H. B. Giraldelo L. A. Santos I. B. Esteves S. N. (2020). Four Single Nucleotide Polymorphisms (SNPs) Are Associated with Resistance and Resilience to *Haemonchus contortus* in Brazilian Morada Nova Sheep. Vet. Parasitol. 279, 109053. 10.1016/j.vetpar.2020.109053 32109653

[B40] Hajishengallis G. Chavakis T. (2019). DEL-1-Regulated Immune Plasticity and Inflammatory Disorders. Trends Molecular Medicine 25 (5), 444–459. 10.1016/j.molmed.2019.02.010 PMC6488420 30885428

[B41] Hanayama R. Tanaka M. Miwa K. Nagata S. (2004). Expression of Developmental Endothelial Locus-1 in a Subset of Macrophages for Engulfment of Apoptotic Cells. J. Immunol. 172 (6), 3876–3882. Baltimore, Md. 1950. 10.4049/jimmunol.172.6.3876 15004195

[B42] Hassan N. M. F. Aboelsoued D. Farag T. K. Hassan S. E. Abu El Ezz N. M. T. (2019). Assessment of *Haemonchus contortus* Larval and Adult Somatic Antigens in Sero-Diagnosis of Haemonchosis in Naturally Infected Sheep and Goats. J. Parasit Dis. 43 (4), 718–725. 10.1007/s12639-019-01152-0 31749544 PMC6841837

[B43] Henderson Y. C. Chou M. Deisseroth A. B. (1997). Interferon Regulatory Factor 1 Induces the Expression of the Interferon-Stimulated Genes. Br. J. Haematol. 96 (3), 566–575. 10.1046/j.1365-2141.1997.d01-2057.x 9054665

[B44] Herrmann F. Trowsdale J. Huber C. Seliger B. (2003). Cloning and Functional Analyses of the Mouse Tapasin Promoter. Immunogenetics 55 (6), 379–388. 10.1007/s00251-003-0597-2 12942211

[B45] Hess J. Angel P. Schorpp-Kistner M. (2004). AP-1 Subunits: Quarrel and harmony Among Siblings. J. Cel. Sci. 117 (Pt 25), 5965–5973. 10.1242/jcs.01589 15564374

[B46] Hollander M. Wolfe D. A. (1973). Nonparametric Statistical Methods. John Wiley and Sons Perry. Wolff 1074, 156–158.

[B47] Honda K. Taniguchi T. (2006). IRFs: Master Regulators of Signalling by Toll-like Receptors and Cytosolic Pattern-Recognition Receptors. Nat. Rev. Immunol. 6 (9), 644–658. 10.1038/nri1900 16932750

[B48] Jiang M. C. Ni J. J. Cui W. Y. Wang B. Y. Zhuo W. (2019). Emerging Roles of lncRNA in Cancer and Therapeutic Opportunities. Am. J. Cancer Res. 9 (7), 1354–1366. 31392074 PMC6682721

[B49] Kang H. M. Sul J. H. Service S. K. Zaitlen N. A. Kong S.-y. Freimer N. B. (2010). Variance Component Model to Account for Sample Structure in Genome-wide Association Studies. Nat. Genet. 42 (4), 348–354. 10.1038/ng.548 20208533 PMC3092069

[B50] Kano S.-i. Sato K. Morishita Y. Vollstedt S. Kim S. Bishop K. (2008). The Contribution of Transcription Factor IRF1 to the Interferon-γ-Interleukin 12 Signaling axis and TH1 versus TH-17 Differentiation of CD4+ T Cells. Nat. Immunol. 9 (1), 34–41. 10.1038/ni1538 18059273

[B51] Karrow N. A. Goliboski K. Stonos N. Schenkel F. Peregrine A. (2014). Review: Genetics of Helminth Resistance in Sheep. Can. J. Anim. Sci. 94 (1), 1–9. 10.4141/cjas2013-036

[B52] Kassambara A. (2020). Ggpubr: 'ggplot2' Based Publication Ready Plots. R package version 0.4.0 Available at: https://CRAN.R-project.org/package=ggpubr .

[B53] Keane O. M. Hanrahan J. P. McRae K. M. Good B. (2018). An Independent Validation Study of Loci Associated with Nematode Resistance in Sheep. Anim. Genet. 49 (3), 265–268. 10.1111/age.12649 29570808

[B54] Kemper K. E. Emery D. L. Bishop S. C. Oddy H. Hayes B. J. Dominik S. (2011). The Distribution of SNP Marker Effects for Faecal Worm Egg Count in Sheep, and the Feasibility of Using These Markers to Predict Genetic merit for Resistance to Worm Infections. Genet. Res. 93 (3), 203–219. 10.1017/S0016672311000097 24725775

[B55] Kijas J. W. Porto-Neto L. Dominik S. Reverter A. Bunch R. McCulloch R. International Sheep Genomics Consortium (2014). Linkage Disequilibrium over Short Physical Distances Measured in Sheep Using a High-Density SNP Chip. Anim. Genet. 45 (5), 754–757. 10.1111/age.12197 25040320

[B56] Kotze A. C. Prichard R. K. (2016). Anthelmintic Resistance in *Haemonchus contortus* . Adv. Parasitol. 93, 397–428. 10.1016/bs.apar.2016.02.012 27238009

[B57] Kourtzelis I. Li X. Mitroulis I. Grosser D. Kajikawa T. Wang B. (2019). DEL-1 Promotes Macrophage Efferocytosis and Clearance of Inflammation. Nat. Immunol. 20 (1), 40–49. 10.1038/s41590-018-0249-1 30455459 PMC6291356

[B58] Li M. Zhong D. Li G. (2021). Regulatory Role of Local Tissue Signal Del-1 in Cancer and Inflammation: a Review. Cell Mol Biol Lett 26 (1), 31. 10.1186/s11658-021-00274-9 34217213 PMC8254313

[B59] Liang Y. Zhou Y. Shen P. (2004). NF-kappaB and its Regulation on the Immune System. Cell Mol Immunol 1 (5), 343–350. 16285893

[B60] MacKinnon K. M. Burton J. L. Zajac A. M. Notter D. R. (2009). Microarray Analysis Reveals Difference in Gene Expression Profiles of Hair and Wool Sheep Infected with *Haemonchus contortus* . Vet. Immunol. immunopathology 130 (3-4), 210–220. 10.1016/j.vetimm.2009.02.013 19346008

[B61] McRae K. M. McEwan J. C. Dodds K. G. Gemmell N. J. (2014). Signatures of Selection in Sheep Bred for Resistance or Susceptibility to Gastrointestinal Nematodes. BMC Genomics 15, 637. 10.1186/1471-2164-15-637 25074012 PMC4124167

[B62] McRae K. M. Stear M. J. Good B. Keane O. M. (2015). The Host Immune Response to Gastrointestinal Nematode Infection in Sheep. Parasite Immunol. 37 (12), 605–613. 10.1111/pim.12290 26480845 PMC4744952

[B63] Miller J. E. Horohov D. W. (2006). Immunological Aspects of Nematode Parasite Control in Sheep1. J. Anim. Sci. 84 (Suppl. l), E124–E132. 10.2527/2006.8413_supple124x 16582083

[B64] Mitroulis I. Chen L.-S. Singh R. P. Kourtzelis I. Economopoulou M. Kajikawa T. (2017). Secreted Protein Del-1 Regulates Myelopoiesis in the Hematopoietic Stem Cell Niche. J. Clin. Invest. 127 (10), 3624–3639. 10.1172/JCI92571 28846069 PMC5617665

[B65] Naeem M. Iqbal Z. Roohi N. (2020). Ovine Haemonchosis: a Review. Trop. Anim. Health Prod. 53 (1), 19. 10.1007/s11250-020-02439-8 33216230 PMC7677603

[B66] NCBI Resource Coordinators (2016). Database Resources of the National Center for Biotechnology Information. Nucleic Acids Res. 44 (D1), D7–D19. 10.1093/nar/gkv1290 26615191 PMC4702911

[B67] Ngere L. Burke J. M. Morgan J. L. M. Miller J. E. Notter D. R. (2018). Genetic Parameters for Fecal Egg Counts and Their Relationship with Body Weights in Katahdin Lambs. J. Anim. Sci. 96 (5), 1590–1599. 10.1093/jas/sky064 29635633 PMC6140914

[B68] Notter D. R. Andrew S. A. Zajac A. M. (2003). Responses of Hair and Wool Sheep to a Single Fixed Dose of Infective Larvae of *Haemonchus contortus* . Small Ruminant Res. 47 (3), 221–225. 10.1016/S0921-4488(02)00279-1

[B69] Notter D. R. Burke J. M. Miller J. E. Morgan J. L. M. (2017). Factors Affecting Fecal Egg Counts in Periparturient Katahdin Ewes and Their Lambs. J. Anim. Sci. 95 (1), 103–112. 10.2527/jas.2016.095510.2527/jas2016.0955 28177372

[B70] Pahl H. L. (1999). Activators and Target Genes of Rel/NF-Κb Transcription Factors. Oncogene 18 (49), 6853–6866. 10.1038/sj.onc.1203239 10602461

[B71] Papadopoulos E. Gallidis E. Ptochos S. (2012). Anthelmintic Resistance in Sheep in Europe: a Selected Review. Vet. Parasitol. 189 (1), 85–88. 10.1016/j.vetpar.2012.03.036 22503039

[B72] Ploeger H. W. Everts R. R. (2018). Alarming Levels of Anthelmintic Resistance against Gastrointestinal Nematodes in Sheep in the Netherlands. Vet. Parasitol. 262, 11–15. 10.1016/j.vetpar.2018.09.007 30389005

[B73] Pollott G. E. Greeff J. C. (2004). Genotype × Environment Interactions and Genetic Parameters for Fecal Egg Count and Production Traits of Merino Sheep1. J. Anim. Sci. 82 (10), 2840–2851. 10.2527/2004.82102840x 15484934

[B74] Purcell S. Neale B. Todd-Brown K. Thomas L. Ferreira M. A. R. Bender D. (2007). PLINK: a Tool Set for Whole-Genome Association and Population-Based Linkage Analyses. Am. J. Hum. Genet. 81 (3), 559–575. 10.1086/519795 17701901 PMC1950838

[B75] R Core Team (2020). R: A Language and Environment for Statistical Computing. Vienna, Austria: R Foundation for Statistical Computing. URL https://www.R-project.org/ .

[B76] Ray N. Kuwahara M. Takada Y. Maruyama K. Kawaguchi T. Tsubone H. (2006). c-Fos Suppresses Systemic Inflammatory Response to Endotoxin. Int. Immunol. 18 (5), 671–677. 10.1093/intimm/dxl004 16569682

[B77] Reuter S. Gupta S. C. Chaturvedi M. M. Aggarwal B. B. (2010). Oxidative Stress, Inflammation, and Cancer: How Are They Linked. Free Radic. Biol. Med. 49 (11), 1603–1616. 10.1016/j.freeradbiomed.2010.09.006 20840865 PMC2990475

[B78] Riggio V. Matika O. Pong-Wong R. Stear M. J. Bishop S. C. (2013). Genome-wide Association and Regional Heritability Mapping to Identify Loci Underlying Variation in Nematode Resistance and Body Weight in Scottish Blackface Lambs. Heredity 110 (5), 420–429. 10.1038/hdy.2012.90 23512009 PMC3630823

[B79] Roeber F. Jex A. R. Gasser R. B. (2013). Next-Generation Molecular-Diagnostic Tools for Gastrointestinal Nematodes of Livestock, with an Emphasis on Small Ruminants. Adv. Parasitol. 83, 267–333. 10.1016/B978-0-12-407705-8.00004-5 23876874 PMC7150098

[B80] Roebuck K. A. (1999). Oxidant Stress Regulation of IL-8 and ICAM-1 Gene Expression: Differential Activation and Binding of the Transcription Factors AP-1 and NF-kappaB (Review). Int. J. Mol. Med. 4 (3), 223–230. 10.3892/ijmm.4.3.223 10425270

[B81] Rouillard A. D. Gundersen G. W. Fernandez N. F. Wang Z. Monteiro C. D. McDermott M. G. (2016). The Harmonizome: a Collection of Processed Datasets Gathered to Serve and Mine Knowledge about Genes and Proteins. Database 2016, baw100. 10.1093/database/baw100 27374120 PMC4930834

[B82] Sallé G. Jacquiet P. Gruner L. Cortet J. Sauvé C. Prévot F. (2012). A Genome Scan for QTL Affecting Resistance to *Haemonchus contortus* in Sheep1. J. Anim. Sci. 90 (13), 4690–4705. 10.2527/jas.2012-5121 22767094

[B83] Sandelin A. Wasserman W. W. Lenhard B. (2004). ConSite: Web-Based Prediction of Regulatory Elements Using Cross-Species Comparison. Nucleic Acids Res. 32, W249–W252. Web Server issueW252. 10.1093/nar/gkh372 15215389 PMC441510

[B84] Sehrawat S. Rouse B. T. (2017). Interplay of Regulatory T Cell and Th17 Cells during Infectious Diseases in Humans and Animals. Front. Immunol. 8, 341. 10.3389/fimmu.2017.00341 28421070 PMC5377923

[B85] Shaulian E. Karin M. (2002). AP-1 as a Regulator of Cell Life and Death. Nat. Cel Biol 4 (5), E131–E136. 10.1038/ncb0502-e131 11988758

[B86] Shaw R. J. Morris C. A. Wheeler M. Tate M. Sutherland I. A. (2012). Salivary IgA: a Suitable Measure of Immunity to Gastrointestinal Nematodes in Sheep. Vet. Parasitol. 186 (1-2), 109–117. 10.1016/j.vetpar.2011.11.051 22153121

[B87] Shin J. Hosur K. B. Pyaram K. Jotwani R. Liang S. Chavakis T. (20132013). Expression and Function of the Homeostatic Molecule Del-1 in Endothelial Cells and the Periodontal Tissue. Clin. Dev. Immunol. 2013, 1–12. 10.1155/2013/617809 PMC3876683 24416060

[B88] SNP & Variation Suite ™ (1998) (Version 8.9.0) [Software]. Bozeman, MT: Golden Helix, Inc. Available at: http://www.goldenhelix.com .

[B89] Son Y.-H. Jeong Y.-T. Lee K.-A. Choi K.-H. Kim S.-M. Rhim B.-Y. (2008). Roles of MAPK and NF-Κb in Interleukin-6 Induction by Lipopolysaccharide in Vascular Smooth Muscle Cells. J. Cardiovasc. Pharmacol. 51 (1), 71–77. 10.1097/FJC.0b013e31815bd23d 18209571

[B90] Stewart W. H. I. T. Scott D. Howell S. Kaplan R. Roeder B. Murphy T. (2020). Anthelmintic Resistance in Gastrointestinal Nematodes and Associated Management Factors in Intermountain West Sheep Flocks. Sheep Goat Res. J. 35, 1–4.

[B91] Suchý T. Zieschang C. Popkova Y. Kaczmarek I. Weiner J. Liebing A.-D. Çakir M. V. Landgraf K. Gericke M. Pospisilik J. A. Körner A. Heiker J. T. Dannenberger D. Schiller J. Schöneberg T. Liebscher I. Thor D. (2020). The Repertoire of Adhesion G Protein-Coupled Receptors in Adipocytes and Their Functional Relevance. Int. J. Obes. 44 (10), 2124–2136. 10.1038/s41366-020-0570-2 PMC7508673 32203115

[B92] Szalóki N. Krieger J. W. Komáromi I. Tóth K. Vámosi G. (2015). Evidence for Homodimerization of the C-Fos Transcription Factor in Live Cells Revealed by Fluorescence Microscopy and Computer Modeling. Mol. Cel Biol 35 (21), 3785–3798. 10.1128/MCB.00346-15 PMC4589601 26303532

[B93] Taki S. Sato T. Ogasawara K. Fukuda T. Sato M. Hida S. (1997). Multistage Regulation of Th1-type Immune Responses by the Transcription Factor IRF-1. Immunity 6 (6), 673–679. 10.1016/s1074-7613(00)80443-4 9208840

[B94] Taniguchi T. Ogasawara K. Takaoka A. Tanaka N. (2001). IRF Family of Transcription Factors as Regulators of Host Defense. Annu. Rev. Immunol. 19, 623–655. 10.1146/annurev.immunol.19.1.623 11244049

[B95] The RNAcentral Consortium (2019). RNAcentral: a Hub of Information for Non-coding RNA Sequences. Nucleic Acids Res. 47, D1250–D1251. Issue D1, D221, 08 January 2019. 10.1093/nar/gky120610.1093/nar/gky1034 30535383 PMC6323998

[B96] Thorne J. W. Murdoch B. M. Freking B. A. Redden R. R. Murphy T. W. Taylor J. B. (2021). Evolution of the Sheep Industry and Genetic Research in the United States: Opportunities for Convergence in the Twenty‐first century. Anim. Genet. 52 (4), 395–408. 10.1111/age.13067 33955573 PMC8360125

[B97] Toscano J. H. B. Okino C. H. Dos Santos I. B. Giraldelo L. A. von Haehling M. B. Esteves S. N. (2019). Innate Immune Responses Associated with Resistance againstHaemonchus Contortusin Morada Nova Sheep. J. Immunol. Res. 2019, 1–10. 10.1155/2019/3562672 PMC6877983 31815153

[B98] Vallabhapurapu S. Karin M. (2009). Regulation and Function of NF-Κb Transcription Factors in the Immune System. Annu. Rev. Immunol. 27, 693–733. 10.1146/annurev.immunol.021908.132641 19302050

[B99] Vanimisetti H. B. Greiner S. P. Zajac A. M. Notter D. R. (2004). Performance of Hair Sheep Composite Breeds: Resistance of Lambs to *Haemonchus contortus*1. J. Anim. Sci. 82 (2), 595–604. 10.2527/2004.822595x 14974560

[B100] Weir B. S. (1996). Genetic Data Analysis II. Sunderland. MA: Sinauer Associates, 161–173.

[B101] Wildeus S. (1997). Hair Sheep Genetic Resources and Their Contribution to Diversified Small Ruminant Production in the United States. J. Anim. Sci. 75 (3), 630–640. 10.2527/1997.753630x 9078477

[B102] Woolaston R. R. Piper L. R. (1996). Selection of Merino Sheep for Resistance to *Haemonchus contortus*: Genetic Variation. Anim. Sci. 62 (3), 451–460. 10.1017/S1357729800014995

[B103] Wu Z. Liu X. Liu L. Deng H. Zhang J. Xu Q. (2014). Regulation of lncRNA Expression. Cell Mol. Biol. Lett. 19 (4), 561–575. 10.2478/s11658-014-0212-6 25311814 PMC6275606

[B104] Xie X. Rigor P. Baldi P. (2009). MotifMap: a Human Genome-wide Map of Candidate Regulatory Motif Sites. Bioinformatics (Oxford, England) 25 (2), 167–174. 10.1093/bioinformatics/btn605 PMC2732295 19017655

[B105] Yan S. Chen L. Zhao Q. Liu Y.-N. Hou R. Yu J. (2018). Developmental Endothelial Locus-1 (Del-1) Antagonizes Interleukin-17-Mediated Allergic Asthma. Immunol. Cel Biol 96 (5), 526–535. 10.1111/imcb.12023 29437247

[B106] Zajac A. M. (2006). Gastrointestinal Nematodes of Small Ruminants: Life Cycle, Anthelmintics, and Diagnosis. Vet. Clin. North America: Food Anim. Pract. 22 (3), 529–541. 10.1016/j.cvfa.2006.07.006 17071351

