# Supplementary material for: Variants Within Genes EDIL3 and ADGRB3 are Associated With Divergent Fecal Egg Counts in Katahdin Sheep at Weaning
Source: Front Genet. 2022 Mar 10;13:817319. doi: 10.3389/fgene.2022.817319 (PMC8960952; doi:10.3389/fgene.2022.817319)
Supplement: Supplementary file 1 [file Presentation1.pdf]

Supplementary Material

1 Supplementary Data

2 Supplementary Figures and Tables

2.1 Supplementary Figures

Supplemental Figures

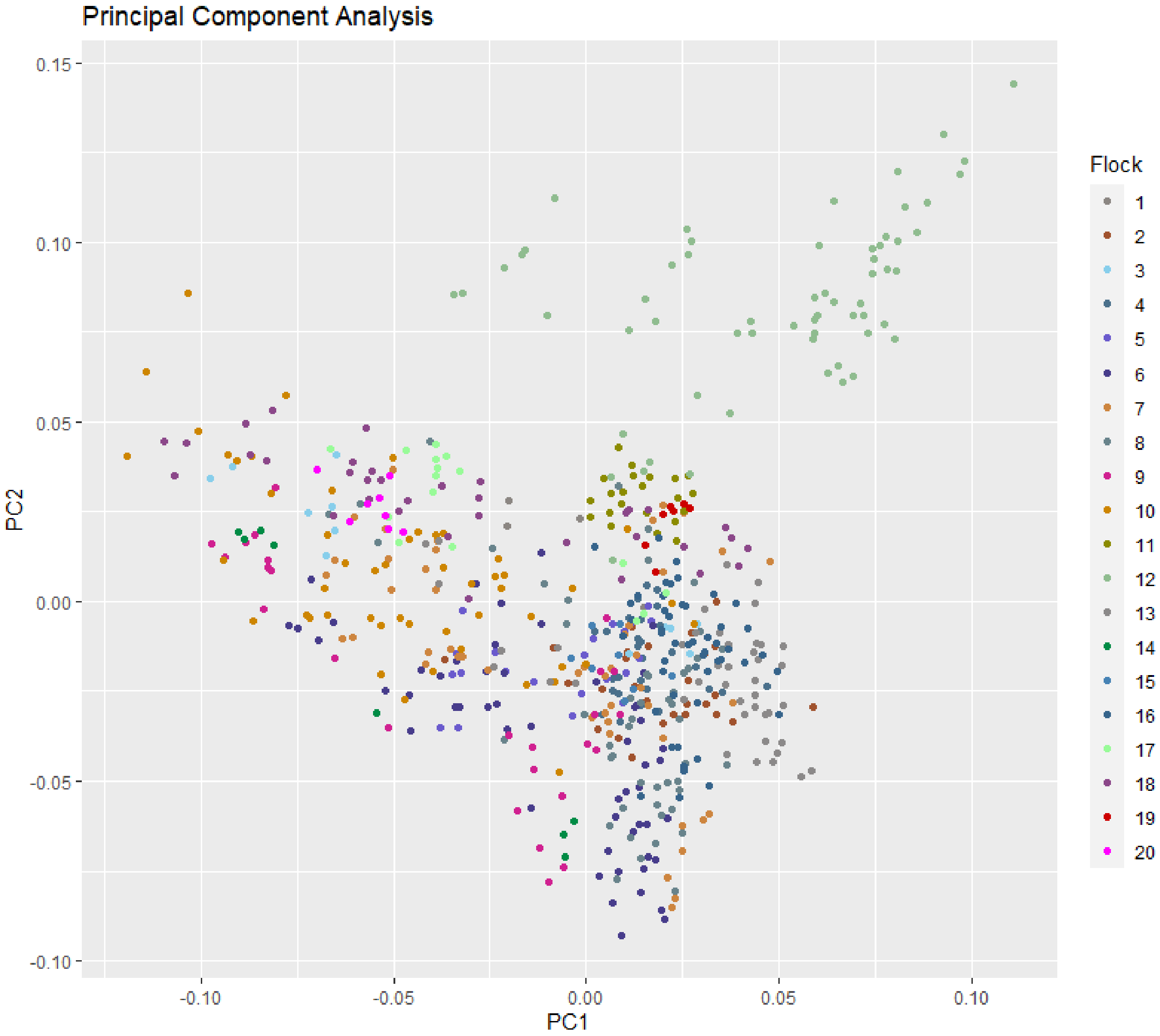

Supplementary Figure S1. Principal component analysis (PCA) of Katahdin study population. Animals are color-coded by flock.

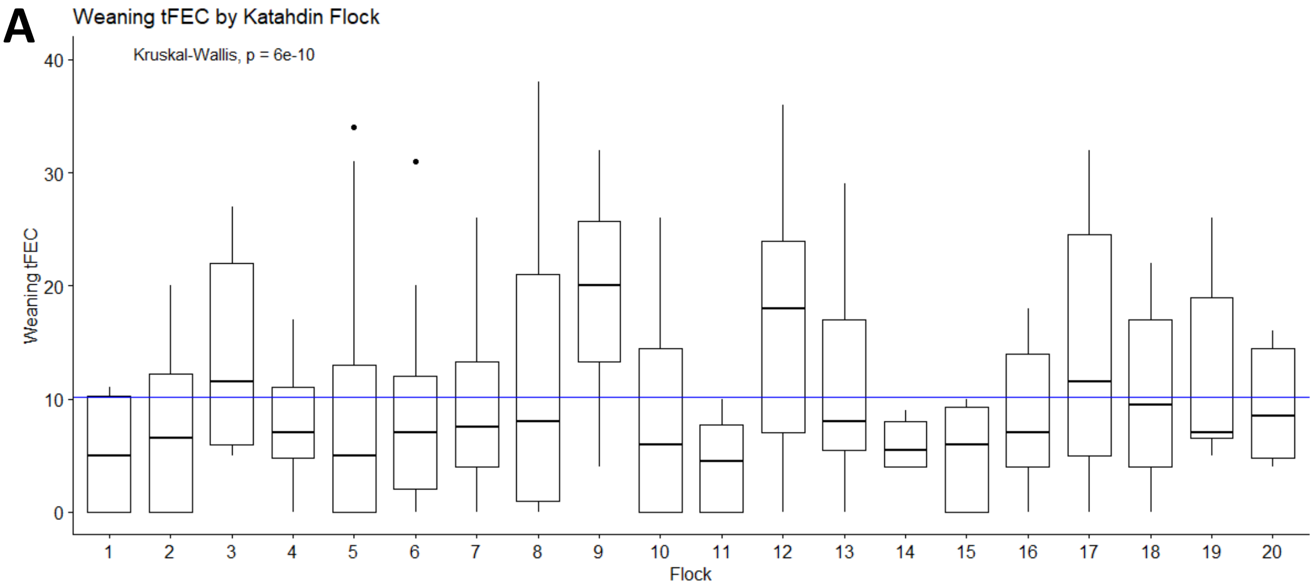

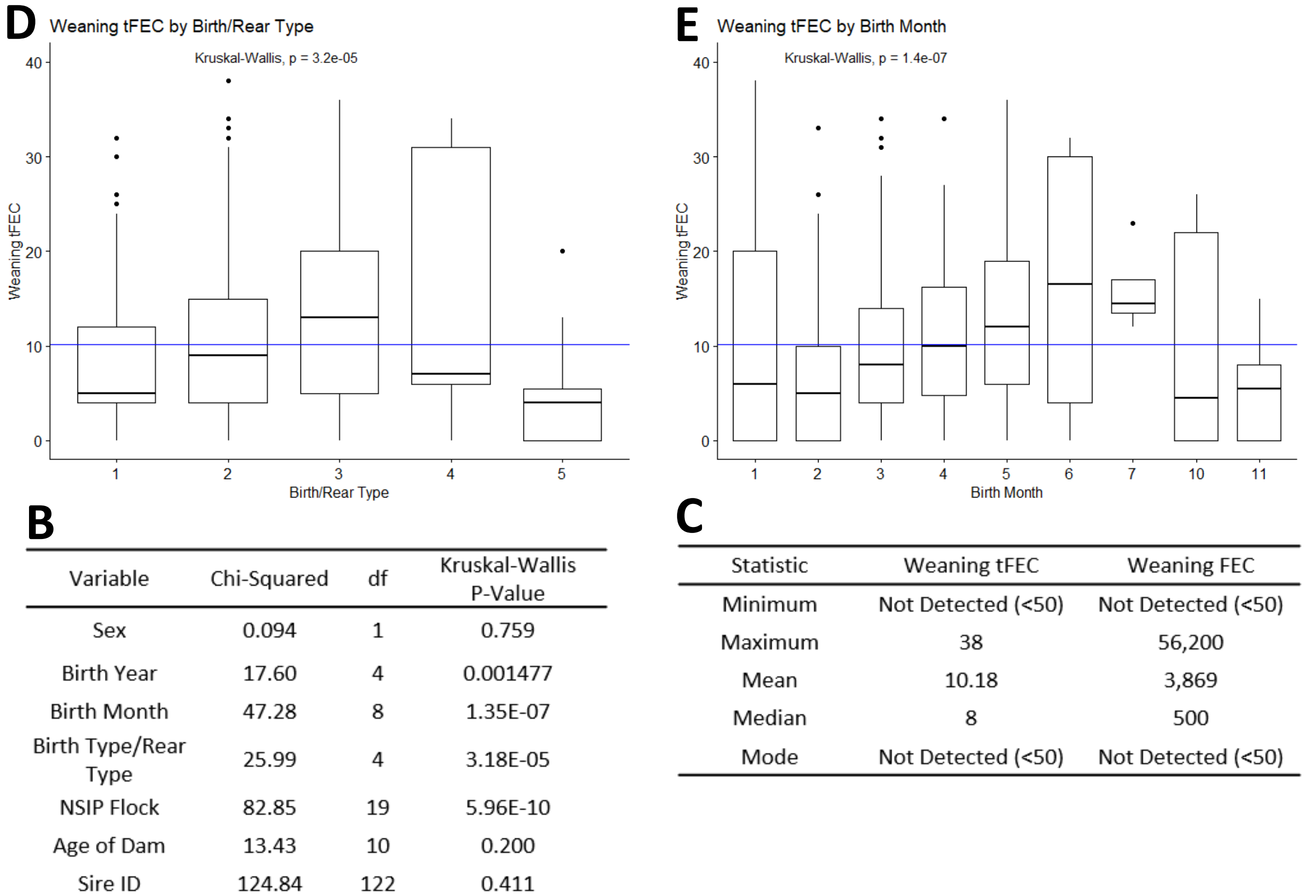

B

Variable	Chi-Squared	df	Kruskal-Wallis P-Value
Sex	0.094	1	0.759
Birth Year	17.60	4	0.001477
Birth Month	47.28	8	1.35E-07
Birth Type/Rear Type	25.99	4	3.18E-05
NSIP Flock	82.85	19	5.96E-10
Age of Dam	13.43	10	0.200
Sire ID	124.84	122	0.411

C

Statistic	Weaning tFEC	Weaning FEC
Minimum	Not Detected (<50)	Not Detected (<50)
Maximum	38	56,200
Mean	10.18	3,869
Median	8	500
Mode	Not Detected (<50)	Not Detected (<50)

Supplementary Figure S2. Statistical evaluation of potential GWAS fixed effects against weaning tFEC. **(**A) Katahdin NSIP-enrolled flock and weaning tFEC, (B) birth/rear type and weaning tFEC,

(C) birth month and weaning tFEC, (D) summary table of Kruskal-Wallis testing of significant and non-significant variables, (E) summary of weaning tFEC and FEC phenotypic data. The horizontal blue line in A, B and C represents the population mean weaning tFEC.

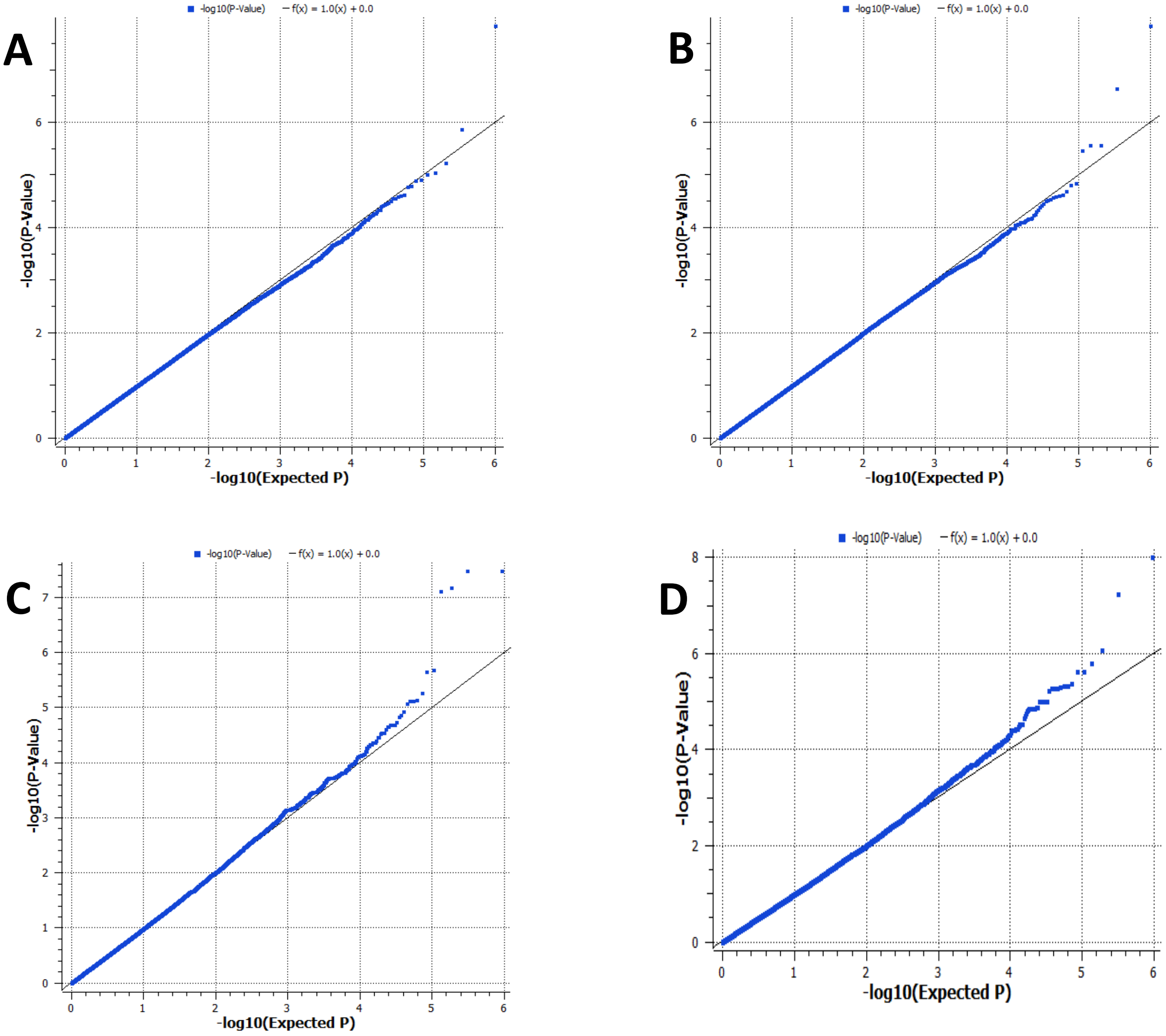

Supplementary Figure S3. Quantile-quantile (QQ) plot from linear regression (LR) and case-control (CC) EMMAX GWAS with weaning tFEC. QQ plots displaying observed versus expected *P*-values

for EMMAX GWAS for (A) LR additive inheritance model, (B) LR dominant inheritance model, (C) LR recessive inheritance model and (D) CC recessive model.

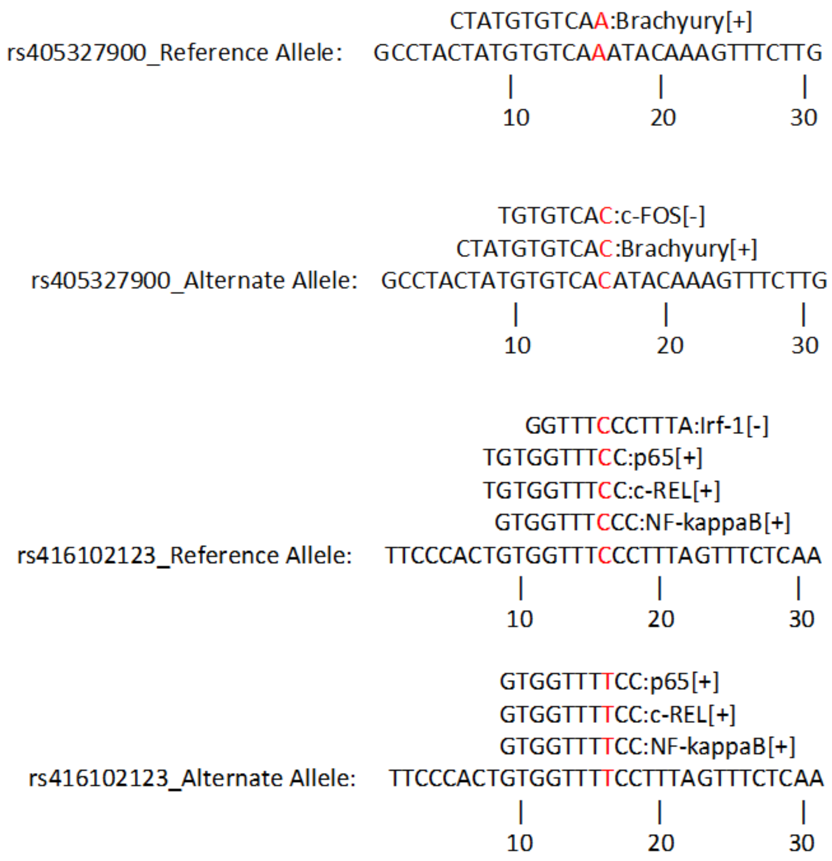

Supplementary Figure S4. Graphical representation of ConSite transcription factor binding site (TFBS) analysis. Figure displays sequence alignment results for TFBS analysis. Results with score differences between alternate and reference allele sequences are displayed.

Supplementary Table S1. ConSite transcription factor binding site (TFBS) analysis results. Table displays complete results from ConSite query of significant SNPs. The 15 bp immediately 3' and 5' of each SNP were tested with either the reference or alternate SNP allele in position 16 of the query sequence. No results are shown for rs417983470 as sequence queries did not predict any TFBS when tested with alternate or reference allele.

DNA Sequence with SNP Reference Allele						DNA Sequence with SNP Alternate Allele					
**rs405327900**											
**Transcription factor**	**Sequence**	**From**	**To**	**Score**	**Strand**	**Transcription factor**	**Sequence**	**From**	**To**	**Score**	**Strand**
Brachyury	CTATGTGTCAA	6	16	12.307	+	Brachyury	CTATGTGTCAC	6	16	9.999	+
Broad-complex_1	TATGTGTCAAATAC	7	20	8.514	-	c-FOS	ATGTGTCA	8	15	6.529	+
c-FOS	ATGTGTCA	8	15	6.529	+	c-FOS	TGTGTCAC	9	16	8.141	-
HFH-1	CAAAGTTTCTT	20	30	7.786	+	HFH-1	CAAAGTTTCTT	20	30	7.786	+
**rs413712238**											
**Transcription factor**	**Sequence**	**From**	**To**	**Score**	**Strand**	**Transcription factor**	**Sequence**	**From**	**To**	**Score**	**Strand**
Thing1-E47	TTTCTGTAAT	6	15	6.663	+	Thing1-E47	TTTCTGTAAT	6	15	6.663	+
E4BP4	TTCTGTAATAG	7	17	9.032	+	E4BP4	TTCTGTAATGG	7	17	9.032	+
Irf-1	GCTTTCAGCTTT	17	28	9.852	-	Irf-1	GCTTTCAGCTTT	17	28	9.852	-
**rs416102123**											
**Transcription factor**	**Sequence**	**From**	**To**	**Score**	**Strand**	**Transcription factor**	**Sequence**	**From**	**To**	**Score**	**Strand**
SOX17	CCCACTGTG	3	11	6.855	+	SOX17	CCCACTGTG	3	11	6.855	+
AML-1	ACTGTGGTT	6	14	11.349	+	AML-1	ACTGTGGTT	6	14	11.349	+
Dorsal_1	CTGTGGTTTCCC	7	18	7.992	+	Dorsal_1	TGTGGTTTTCCT	8	19	7.766	+
Dorsal_2	GTGGTTTCCC	9	18	10.489	+	Dorsal_2	GTGGTTTTCC	9	18	12.811	+
NF-kappaB	GTGGTTTCCC	9	18	7.701	+	NF-kappaB	GTGGTTTTCC	9	18	7.849	+
c-REL	TGTGGTTTCC	8	17	8.335	+	c-REL	GTGGTTTTCC	9	18	9.179	+
P65	TGTGGTTTCC	8	17	7.822	+	P65	GTGGTTTTCC	9	18	9.132	+
Irf-1	GGTTTCCCTTTA	11	22	10.652	-	E74A	TTTCCTT	14	20	6.669	-
